# Bystander Activation and Anti-Tumor Effects of CD8+ T Cells Following Interleukin-2 Based Immunotherapy Is Independent of CD4+ T Cell Help

**DOI:** 10.1371/journal.pone.0102709

**Published:** 2014-08-13

**Authors:** Arta M. Monjazeb, Julia K. Tietze, Steven K. Grossenbacher, Hui-Hua Hsiao, Anthony E. Zamora, Annie Mirsoian, Brent Koehn, Bruce R. Blazar, Jonathan M. Weiss, Robert H. Wiltrout, Gail D. Sckisel, William J. Murphy

**Affiliations:** 1 Department of Radiation Oncology School of Medicine, University of California Davis, Sacramento, California, United States of America; 2 Department of Dermatology, School of Medicine, University of California Davis, Sacramento, California, United States of America; 3 Department of Pediatrics, Division of Blood and Marrow Transplantation and Masonic Cancer Center, University of Minnesota, Minneapolis, Massachusetts, United States of America; 4 Cancer and Inflammation Program, National Cancer Institute, Frederick, Maryland, United States of America; 5 Department of Internal Medicine, School of Medicine, University of California, Davis, Sacramento, California, United States of America; Baylor College of Medicine, United States of America

## Abstract

We have previously demonstrated that immunotherapy combining agonistic anti-CD40 and IL-2 (IT) results in synergistic anti-tumor effects. IT induces expansion of highly cytolytic, antigen-independent “bystander-activated” (CD8^+^CD44^high^) T cells displaying a CD25^−^NKG2D^+^ phenotype in a cytokine dependent manner, which were responsible for the anti-tumor effects. While much attention has focused on CD4+ T cell help for antigen-specific CD8+ T cell expansion, little is known regarding the role of CD4+ T cells in antigen-nonspecific bystander-memory CD8+ T cell expansion. Utilizing CD4 deficient mouse models, we observed a significant expansion of bystander-memory T cells following IT which was similar to the non-CD4 depleted mice. Expanded bystander-memory CD8+ T cells upregulated PD-1 in the absence of CD4+ T cells which has been published as a hallmark of exhaustion and dysfunction in helpless CD8+ T cells. Interestingly, compared to CD8+ T cells from CD4 replete hosts, these bystander expanded cells displayed comparable (or enhanced) cytokine production, lytic ability, and in vivo anti-tumor effects suggesting no functional impairment or exhaustion and were enriched in an effector phenotype. There was no acceleration of the post-IT contraction phase of the bystander memory CD8+ response in CD4-depleted mice. The response was independent of IL-21 signaling. These results suggest that, in contrast to antigen-specific CD8+ T cell expansion, CD4+ T cell help is not necessary for expansion and activation of antigen-nonspecific bystander-memory CD8+ T cells following IT, but may play a role in regulating conversion of these cells from a central memory to effector phenotype. Additionally, the expression of PD-1 in this model appears to be a marker of effector function and not exhaustion.

## Introduction

Classically, naïve and memory T cell activating signals include engagement of T-cell receptor (TCR) by cognate antigen in the setting of MHC. In a phenomenon termed “bystander activation” memory T-cells can proliferate and activate without the need for antigen specific TCR engagement [1], [2], [3]. These “bystander cells” proliferate and gain effector functions in response to the highly stimulatory local cytokine milieu produced during the course of viral and bacterial infections in mice and humans [4], [5], [6]. The function and regulation of these bystander activated T cells is unclear but they likely play a role in viral clearance [4], [5], [6].

Based on promising results in recent pilot clinical trials for cancer there has been a renewed interest in IL-2 based immunotherapy [7] as well as in agonistic CD40 antibodies [8]. We previously described that a combination immunotherapy consisting of agonist CD40 antibody and high dose systemic IL-2 (IT) resulted in synergistic antitumor effects which were CD8^+^ T-cell dependent [9]. Recently we demonstrated that IT and other strong immunostimulatory therapies can overcome the need for antigen specificity for cytotoxic T lymphocyte (CTL) expansion and tumor cell killing [3]. Such regimens resulted in a massive expansion of CD44^high^ memory, but not naïve, CD8+ T-cells. This “bystander expansion” may play an important role in tumor immunity as it does in viral and bacterial infections. IT-induced bystander CD8+ T cells have a distinct phenotype (CD25^−^NKG2D^+^CD44^high^) from CD8+ T cells activated via T-cell receptor (TCR) engagement and have the ability to initiate effector functions and cell killing independent of TCR engagement. IT-induced CD8+ T cells express NKG2D and provide anti-tumor killing in part due to NKG2D expression [3]. The anti-tumor effects of IT have been observed in a number of murine tumor models but whether this therapy would be effective against a tumor type completely devoid of NKG2D ligands remains unresolved.Further, in models of influenza infection, bystander CD8+ T cells (CD25^−^NKG2D^+^CD44^high^) also acutely expand and play an important role in controlling early viral infection in an antigen nonspecific manner [10]. These findings demonstrate that during conditions of strong immunostimulation, such as viral infection or cancer immunotherapy, there is a massive expansion of cytolytic bystander activated memory phenotype CD8+ T cells which play a critical role in controlling viral infection or tumor in an antigen nonspecific manner.

IT can lead to loss of peripheral CD4+ T cells due to activation-induced cell death [11]. Little is known regarding the role of CD4+ T cells in regulating the expansion and function of bystander activated memory CD8+ T cells. The critical role of CD4+ T cell help in antigen-specific CD8+ T lymphocyte and general immune function is well illustrated by the sequelae suffered by patients suffering from AIDS. The need for CD4+ T-lymphocyte help in the function of both primary and memory CD8+ T lymphocyte responses is well established [12], [13]. It has been demonstrated that the presence of CD4+ help during antigen-specific CD8+ cytotoxic T lymphocyte (CTL) priming is necessary for clonal expansion upon re-encountering antigen, since otherwise the restimulated CD8+ cells undergo TRAIL mediated cell death [14], [15]. Furthermore, despite having been primed in the presence of CD4+ cells, memory CD8+ T cells can become functionally impaired if lacking CD4+ help [16]. Upregulation of PD-1 has become an important hallmark of the exhaustion and dysfunction of “helpless” CD8+ T cells [17], [18]. The importance of CD4+ help has also been demonstrated for the recruitment, proliferation, and effector function of CTLs in the tumor microenvironment [19] and studies demonstrated increased tumor growth after CD4 depletion [20], [21], [22], [23].

To further characterize the immunologic mechanisms behind the anti-tumor effects of IL-2-based immunotherapy and the role of CD4+ T cells in antigen non-specific bystander expansion, we analyzed the phenotype and function of the proliferating CD8+ cells after IT in the absence of CD4+ T cells. We observed that IT induces a massive expansion of CD25^−^NKG2D^+^ bystander memory CD8+ T cells and that the anti-tumor effects are CD8+ dependent. With both the in vivo depletion of CD4+ T cells or the use of CD4 KO mice we observed no change in the function or extent of expansion of CD44^high^ CD8+ T cells displaying a CD25^−^NKG2D^+^ bystander phenotype following immunotherapy compared to IT treated CD4 replete mice. Interestingly, in the absence of CD4^+^ T cells, the expanded bystander activated CD8+ T cell population upregulated PD-1 had an increased effector memory phenotype and did not have any functional evidence of exhaustion. These results suggest that, although antigen non-specific bystander expansion of memory CD8+ T-cells does not require CD4+ help in the same way as antigen specific CD8+ T cell expansion, there is a role for CD4^+^ help in regulating the conversion of these bystander cells from a central memory to effector memory/effector phenotype.

## Materials and Methods

### Ethics Statement

Mouse studies were performed with the approval of the University of California, Davis and University of Minnesota Institutional Animal Care and Use Committees (IACUC). For survival studies mice were sacrificed at humane endpoints as specified by IACUC guidelines using CO2 overdose. Humane endpoints included, but were not limited to, tumor burden greater than or equal to 10% of the animal's normal body weight, tumors exceeding 2 cm in size in, a 20% decrease in body weight, inability to reach food or water, or a body condition score less than 2 on a 5 point scale. Mice were monitored twice daily during the study period. No anesthesia or analgesia was used.

### Mice

C57BL/6, CD4 knockout (B6.129S2-CD4^tm1Mak^/J), and control mice were purchased from the animal production area of the National Cancer Institute (NCI) or The Jackson Laboratory. IL-21 receptor knockout mice (RKO) were generated as previously described [24]. Mice were 8 to 16 weeks old in all studies. Mice were housed in a specific pathogen free facility, four mice per cage, in micro-isolation cages, with a 12 hour light/dark cycle, and free access to food and water.

### IT and depletion regimens

Mice were assigned to treatment or control groups randomly on a cage by cage basis. C57BL/6 mice were treated with agonistic anti-CD40 antibody and recombinant human IL-2 (rhIL-2) or IgG and PBS in the control groups as previously described [9]. The treatment schema is outlined in Fig. S1a. Briefly, anti-CD40 was administered daily for a total of 5 consecutive days (Days: 0, 1, 2, 3 and 4) and IL-2 was administered twice a day for a total of 4 days (Days: 1,4, 8 and 12). Control mice received rat-IgG (rIgG, Jackson ImmunoResearch Laboratories, Inc.) and PBS (Cellgro). Mice received 80 ug of agonist anti-CD40 and 1×10^6^ IU of IL-2 in 0.2 ml PBS i.p. Control mice received 80 ug of rIgG in PBS. The anti-mouse CD40 antibody (clone FGK115B3) was generated via ascites production, as previously described [9]. Recombinant human IL-2 (IL-2; TECIN Teceleukin) was provided by the National Cancer Institute repository (Frederick, MD). For in vivo depletion studies CD4^+^ T cells were depleted with i.p. injections of anti-CD4 antibody at 500 µg (clone GK1.5; gift from G.B. Huffnagle, University of Michigan, Ann Arbor, MI) (Fig. S1a). In short term depletion experiments mice received i.p. injections on days 0, 4, and 8. In long term depletion experiments mice received i.p. injections twice weekly for four weeks. CD8^+^ cells were depleted in vivo by i.p. injection of anti-mouse CD8 (clone 19–178). Two doses of Ab (163 µg/dose) were administered before the beginning of therapy and were continued three times weekly during the course of immunotherapy (>90% depletion). All treatments were performed in the vivarium in the housing cages.

Bromodeoxyuridine (BrdU) was purchased from BD Bioscience (San Jose, CA) and was used per the manufacturer's instructions. In experiments involving BrdU, 1 mg BrdU in 0.1 mL D-PBS was injected intraperitoneally 24 hours prior to harvest.

The 3LL cell line (ATCC) was maintained in RF10 complete media (RF10c). For *in vivo* tumor studies one million 3LL cells were administered by s.c. injection into the flank of C57BL/6 mice. Tumor volume was measured biweekly. All tumor survival experiments contained 8–15 mice/treatment group. In all experiments immunotherapy was initiated 7–10 days after tumor implantation when tumors were roughly 6×6 mm in size.

### Flow cytometry and antibodies

Single cell suspensions were labeled with Fc Block (BD Bioscience) and antibodies for 20 minutes, and then washed twice with staining buffer consisting of DPBS (Mediatech, Herndon, VA) and 1% FBS (Gemini Bio-Products, Sacramento, CA). Samples were analyzed using a custom-configured LSRII with FACSDiva software (Becton Dickinson, San Jose, CA). The IntraPrep kit (Beckman Coulter, Brea, CA) was used for granzyme staining, per manufacturer's instructions. Interferon gamma production was assayed by restimulating splenocytes with PMA/Ionomycin (0.16/1.6 ug/ml) for 4 hours in vitro. Golgi stop (0.7 ug/ml, BD Bioscience) was added following 1 hr of stimulation. Following stimulation, staining and analysis by flow cytometry was performed. Data were analyzed using FlowJo, Version 8 software (TreeStar, Ashland, OR). Antibodies included: PE-Cy7–conjugated anti-CD62L, FITC, PE, PE-Cy5, or APC-conjugated anti-CD25, APC-conjugated anti-CD44, PE or PE-Cy7–conjugated anti-NKG2D, FITC or PE-conjugated anti–PD-1, PE-conjugated anti-Vα2, APC-Cy7–conjugated anti-CD122 (eBioscience, San Diego, CA) FITC or APC-conjugated antiBrdU, APC-conjugated anti-CD8, and APC-Cy7–conjugated anti-CD25 (BD Pharmingen). Pacific Blue–conjugated anti-CD44 (BioLegend, San Diego, CA), PE-TexasRed–conjugated anti-CD8, and PE-conjugated anti–human Granzyme B (Invitrogen, Grand Island, NY). Intracellular staining was performed using staining kits for FoxP3 (eBioscience) and intra-cellular cytokines (BD biosciences) per manufacturer's instructions.

### Antibody-redirected lysis assay

Splenic CD8^+^ T cells were serially diluted in 96-well U bottom plates in RF10c media. P815 (ATCC) cells were labeled with 100uCi ^51^Cr (NEZ030S; Perkin Elmer) per 10^6^ cells and incubated for 30 minutes with 10 ug/mL anti-CD3e (eBiosciences). P815 targets (10^4^) were added to each well and incubated at 37°C for 4 hours. Supernatants were removed, mixed 1∶1 with scintillation fluid, and analyzed on a Wallac scintillation counter (Wallac, Ramsey, MN). Total release was determined by adding 100 uL of 1× Triton X-100 detergent (Sigma-Aldrich, St. Louis, MO) to target cells. Specific release was calculated as: % lysis = 100%×(Experimental-Spontaneous)/(Maximum–Spontaneous).

### Tissue collection and processing

Lymph nodes including the cervical, scapular, axillary, and inguinal nodes were collected at day 11 or day 15 after the initiation of IT. Lymph nodes and spleens were crushed, filtered, and counted in DPBS. Prior to counting, red blood cells were lysed and cells counted using a Z1 Particle Counter (Beckman Coulter).

### Statistics

Statistical analysis was performed using Prism Version 4 (GraphPad Software, Location). For analysis of 3 or more groups, the nonparametric ANOVA test was performed with the Bonferroni post-test. Analysis of differences between 2 normally distributed groups was performed using the Student's *t* test. Nonparametric groups were analyzed with the Mann-Whitney test. Welch's correction was applied to Student's *t* test datasets with significant differences in variance. Data were tested for normality and variance. A *P* value of <0.05 was considered significant (*P<0.05, **P<0.01, ***P<0.001).

## Results

### Expansion of bystander activated CD8+ T cells after systemic immunotherapy (IT)

**Figure 1:**
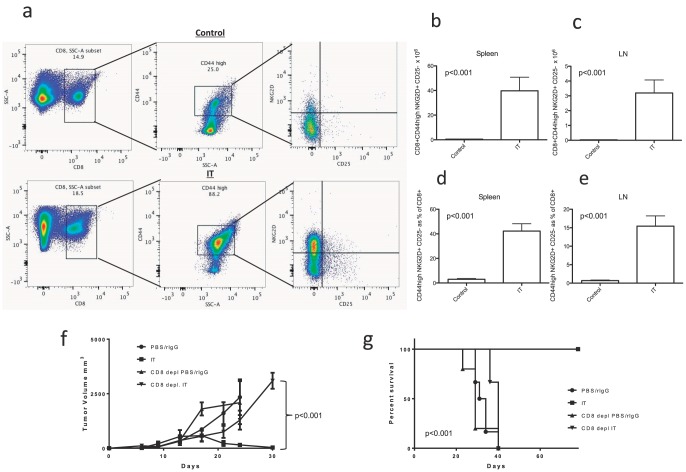
CD40/IL-2 Immunotherapy induces massive expansion of bystander memory CD8+ cells and anti-tumor effects are CD8 dependent. Three C57BL/6 mice per group were treated with IT or PBS/rIgG (control) and effects on CD8+ T cell expansion were quantified by flow cytometric analysis 11 days after the initiation of therapy. For *in vivo* tumor studies one million 3LL cells were administered by s.c. injection into the flank of C57BL/6 mice seven days prior to initiation of therapy. Six to eight 3LL bearing mice were treated with IT and/or CD8+ T cell depletion to examine CD8+ dependence of anti-tumor effects. (**a**) Gating strategy for bystander memory CD8+ CD44^high^ NKG2D+ CD25− cells. (**b–e**) Expansion of bystander memory CD8+ T cells in the spleen and lymph nodes of IT or vehicle treated mice expressed as total numbers (**b,c**) or as a percentage of total CD8+ T cells (**d,e**). Effects of IT and/or CD8 depletion on tumor growth (**f**) and survival (**g**).

The IT treatment schema for high dose IL-2 and agonist anti-CD40 is outlined in Figure S1a. Mice were harvested 11 days after the initiation of therapy to examine immune parameters. IT induced marked expansion of the CD8+ T-cell compartment that was predominately accounted for by the expansion of bystander activated CD8+ T cells with a CD44^high^ NKG2D+ CD25-phenotype (Fig. 1). The population of CD44^high^ T cells in these naïve mice could result from homeostatic expansion or due to encountering cognate antigen in the environment or gut microflora. We have not distinguished between the two and this population which expands in response to therapy could be bona-fide memory or memory-like cells (henceforth referred to simply as memory). The expanded cells are not CD8+ NK cells as they also express CD3 (data not shown) and this phenotype has also been seen on bystander activated OT-1 CD8+ T cells [3]. The number of these bystander activated cells was increased by greater than 30 fold in both the spleen and lymph nodes (Fig. 1b–c, p<0.05). In both the spleen and lymph nodes (LNs) these bystander activated memory cells became a significant portion of the CD8+ T-cell compartment with over 40% of splenic CD8+ T-cells displaying this phenotype after IT (Fig. 1d–e, p<0.05). Additionally, in a 3LL tumor model we found that the anti-tumor effects of IT were abrogated by CD8 depletion illustrating the critical role of CD8+ T-cells in the effectiveness of this therapy. Although IT was able to eradicate tumor growth (Fig. 1f) and lead to long term survival (Fig. 1g), in the absence of CD8+ cells there was no significant difference between untreated and IT treated mice. These effects are likely due to the absence of CD8+ T-cells and not CD8+ NK or DC cells as similar outcomes are seen in SCID mice which lack T-cells but have normal NK and DC cells [9].

### Expansion of bystander activated CD8+ T-cells after IT is independent of CD4+ help

To elucidate the role of CD4+ T cell help in the expansion of bystander activated CD8+ T cells we employed multiple models in which CD4 T cells were absent. A short term (2 week) CD4 depletion model (Figure S1a) was used as the primary model for the majority of experiments. The long term depletion (6 weeks) model was used to confirm our results as it has been suggested that CD8+ T cell dysfunction only occurs after longer periods of lack of CD4 help [16]. Finally, CD4 knockout (CD4 KO) mice were used to validate our results in a model where complete absence of the cells occurs. The number of CD4^+^ T cells in the spleens and LNs of mice in our short term depletion model, long term depletion model and CD4 KO mice were >95% reduced in comparison to control CD4+ replete (CD4+ CTRL) mice (p<0.001, Figure S1b,c). Immunotherapy increased the expansion of the CD4^+^ T cells but CD4 depleted mice still had a significant reduction (p<0.001) in the number of CD4^+^ T cells after IT (Figure S1b,c).

**Figure 2:**
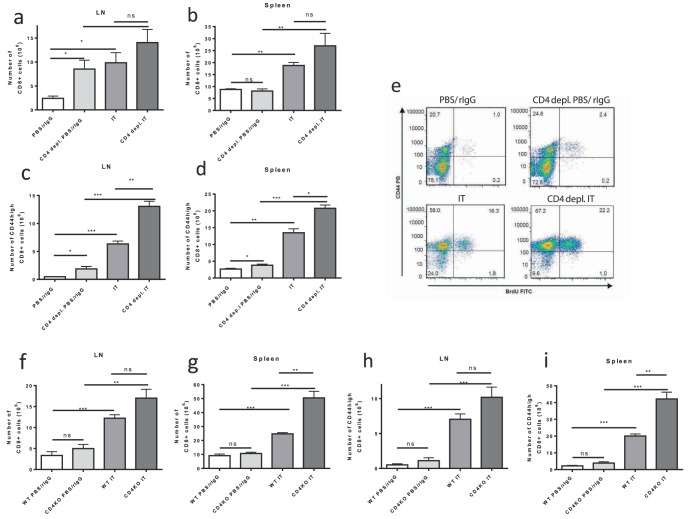
IT induced expansion of memory CD8+ T cells in CD4+ T cell deficient models. Control or CD4 deficient (depleted or knockout) C57BL/6 mice were treated with IT or PBS/rIgG (control) and effects on CD8+ T cell expansion were quantified by flow cytometric analysis 11 days after the initiation of IT. CD8+ (**a,b**) and memory CD8+ (**c,d**) T cell numbers in the LNs (**a,c**) and spleens (**b,d**) of control or CD4+ T cell depleted mice treated with vehicle or IT. (**e**) BrdU incorporation in CD8+ T cells from spleens of control or CD4+ T cell depleted mice treated with vehicle or IT. CD8+ (**f,g**) and memory CD8+ (**h,i**) T cell numbers in the LNs (**f,h**) and spleens (**g,i**) of wild-type or CD4 knockout mice treated with vehicle or IT. Results are representative of two (CD4 knockout) or three (CD4 depletion) independent experiments with a minimum of three mice per group. (*P<.05, **P<.01, ***P<.001).

As demonstrated above, expansion of bystander activated memory CD8+ T cells is observed after treatment of CD4+ CTRL mice with IT (Fig. 1). This expansion can be accompanied by loss of CD4+ T cell help due to activation induced cell death [9], [11]. We questioned whether CD4 depletion may influence the expansion of this antigen-nonspecific bystander memory compartment after IT. The IT induced increase of CD8+ T cells or CD44^high^ memory CD8+ cells in the LNs and spleens of CD4 depleted animals was maintained. In all cases, 11 days after the initiation of therapy IT induced a sizeable and statistically significant increase in cell numbers compared to untreated CD4+ CTRL mice regardless of CD4+ cell status (Fig. 2a–d). Similarly, the percentage of CD8+ or memory CD44^high^ CD8+ T cells after IT was increased in the spleens and LNs of both CD4+ CTRL and CD4 depleted mice compared to vehicle treated mice (data not shown). Additionally, assessment of proliferation by BrdU incorporation demonstrated that the memory CD8+ cells were expanding after IT in both CD4+ CTRL and CD4 depleted mice (Fig. 2e). CD44^high^CD8+ cells were previously observed to proliferate after IT, and this is also the population seen to expand after IT in the setting of CD4 depletion.

The effects of long term CD4 depletion on the memory CD8+ compartment after IT were examined using mice with long term depletion of CD4+ cells starting 30 days prior to treatment, or genetic disruption of CD4, using CD4 KO mice. Mirroring the above results, neither long-term depletion of CD4 (Figure S2) nor CD4 KO mice (Fig. 2f–i) demonstrated a reduction in the IT induced expansion of CD8+ cells or memory CD44^high^CD8+ cells. Hence, in each of our models, that lack of CD4+ cells caused no reduction in the expansion of memory CD8+ T cells in response to IT. Surprisingly, in most instances, regardless of the model examined, the lack of CD4+ T-cells resulted in a trend or a statistically significant increase in the IT-induced expansion of memory CD8+ T cells compared to IT-treated CD4+ CTRL mice (Fig. 2a–d,f–i).

### “Helpless” bystander activated memory CD8+ cells increase expression of PD-1 after IT but do not display functional characteristics of exhaustion and maintain anti-tumor effects in-vivo

**Figure 3:**
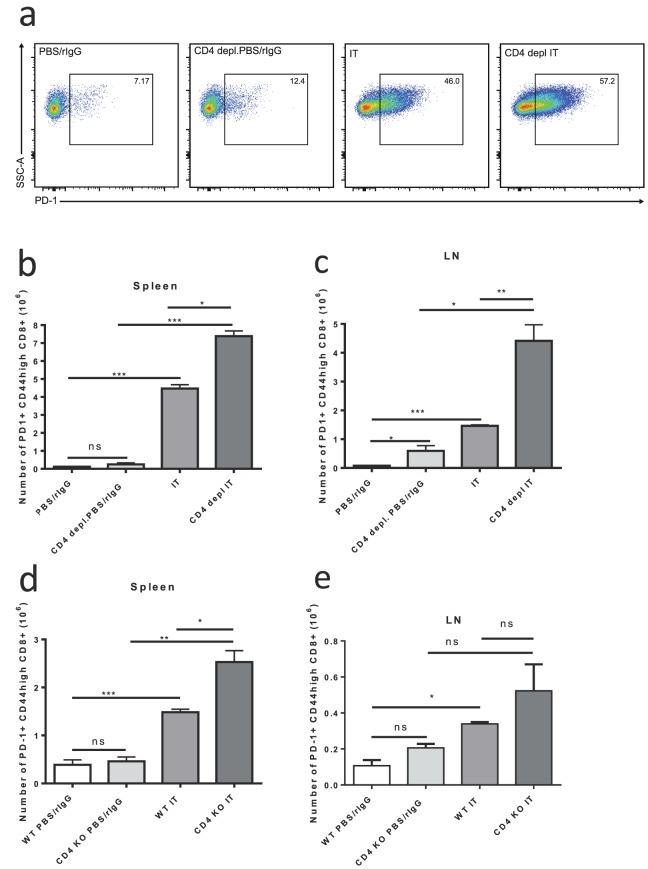
Increased number of PD-1+ memory CD8+ T cells after IT in CD4+ T cell deficient mice. Control or CD4 deficient (depleted or knockout) C57BL/6 mice were treated with IT or PBS/rIgG (control) and PD-1 expression on memory T-cells was quantified by flow cytometric analysis 11 days after the initiation of IT. (**a**) Representative dot plots for PD-1+ gating on CD8+ CD44^high^ cells in the spleens of CD4+ depletion model mice. Number of PD-1+ memory (CD44^high^) CD8+ T cells in spleens (**b,d**) and LNs+ (**c,e**) of IT or vehicle treated mice in CD4+ depletion (**b,c**) or CD4 knockout (**d,e**) models. Results are representative of two (CD4 knockout) or three (CD4 depletion) independent experiments with a minimum of three mice per group. (*P<.05, **P<.01, ***P<.001).

It has been demonstrated in models of viral infection that lack of CD4+ help can upregulate PD-1 expression on CD8+ T cells leading to diminished anti-viral responses and a decrease in central memory CD8+ cells [18], [25]. PD-1 upregulation on CD8+ cells in chronic infections, such as HIV, has been associated with exhaustion of antigen specific CD8 +cells [26]. As demonstrated above, we observed no change in CD8+ T cell expansion after IT despite lack of CD4+ help. We further investigated the role of PD-1 expression after IT in mice lacking CD4 help. Interestingly, we found that, 11 days after the initiation of IT PD-1 expression was increased on memory CD8+ T cells in mice lacking CD4+ T cells compared to IT treated CD4+ CTRL mice (Fig. 3a–e). In CD4+ CTRL, CD4 depleted, and CD4 KO mice the levels of PD-1+ memory CD8+ T cells was minimal at baseline (Fig. 3a–e). IT induced a statistically significant increase in both the percentage (Fig. 3a) and total numbers of PD-1+ memory CD8+ T cells in both CD4+ CTRL and CD4 depleted or KO mice (Fig. 3b–e). Although PD-1 was increased in both the CD4+ CTRL and CD4 deficient models, the increase was most pronounced and significantly higher in the IT-treated CD4 depleted or KO mice when compared to IT treated CD4+ CTRL mice (Fig. 3a–e). For example, the levels of PD-1+ memory CD8+ T cells in the LNs of IT treated CD4 depleted mice were about 3-fold higher than IT-treated control mice (Fig. 3c,p<0.01). Similar results were observed in our long term depletion model (Figure S2).

**Figure 4:**
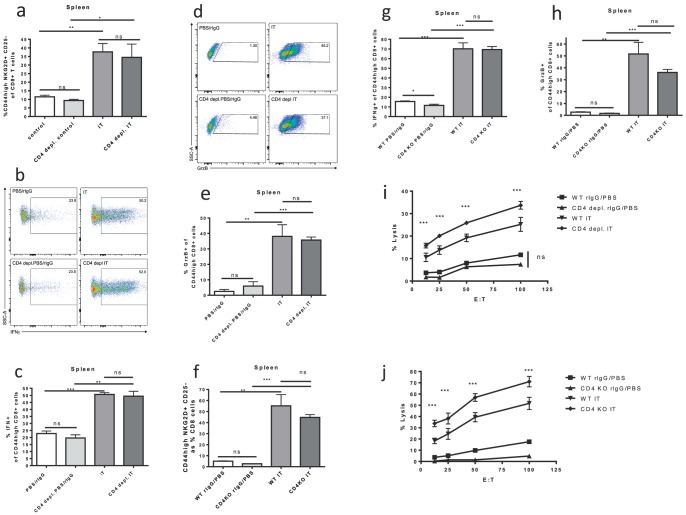
Memory CD8+ T cell function after IT in CD4+ T cell deficient models. Control or CD4 deficient (depleted or knockout) C57BL/6 mice were treated with IT or PBS/rIgG (control) and assessed for function of memory CD8+ T cells 11 days after the initiation of IT. NKG2D and granzyme B expression were quantified by flow cytometric analysis. Interferon gamma production was quantified by flow cytometric analysis after *in vitro* restimulation of splenocytes with PMA/Ionomycin (0.16/1.6 ug/ml) for one hour followed by incubation with golgi stop (0.7 ug/ml) for three hours. CD8+ T cell killing function was assayed by scintillation counting using an *in vitro* redirected lysis assay with ^51^Cr labeled P815 target cells incubated for 30 minutes with 10 ug/mL anti-CD3e. (**a,f**) NKG2D expression on memory CD8+ T cells in CD4 depletion (**a**) and knockout (**f**) models. Representative dot plots for NKG2D+ CD25− gating are presented in Figure 1. (**b**) Representative dot plots for IFNγ+ gating on CD8+ CD44^high^ cells in the spleens of CD4+ depletion model mice. (**c,g**) Interferon gamma production by memory CD8+ T cells in CD4 depletion (**c**) and knockout (**g**) models. (**d**) Representative dot plots for Granzyme B+ gating on CD8+ CD44^high^ cells in the spleens of CD4+ depletion model mice. (**e,h**) Granzyme B expression by memory CD8+ T cells in CD4 depletion (**e**) and knockout (**h**) models. Killing function of splenocytes from CD4 depleted (**i**) or CD knockout (**j**) mice expressed as percentage of maximal lysis. Results are representative of two (CD4 knockout) or three (CD4 depletion) independent experiments with a minimum of three mice per group. (*P<.05, **P<.01, ***P<.001).

Given the intact expansion, but increased exhaustion phenotype of memory CD8+ T cells after IT in CD4 deficient mice we hypothesized that CD4+ help is required for the proper function but not expansion of the bystander memory CD8+ T-cells in response to IL-2 based immunotherapy. To test this hypothesis the phenotype, function, activation and *in-vivo* anti-tumor effects of CD8+ T-cells after IT in the absence of CD4+ cells was investigated. No differences in NKG2D upregulation after IT, which defines the IT induced bystander memory phenotype (CD8+ CD44^high^ NKG2D+ CD25−), was observed between CD4+ CTRL and CD4 depleted (Fig. 4a) or CD4 KO (Fig. 4f) mice 11 days after the initiation of IT. Despite the upregulation of PD-1 in the CD4 deficient models after IT, there was no functional impairment or dysfunction. The ability to produce cytokines was analyzed by assaying the percentage of memory CD8+ T-cells producing IFNγ after *in vitro* stimulation with PMA/Ionomycin. Again, IT significantly increased IFNγ production with no differences between IT treated CD4+ CTRL and CD4 depleted (Fig. 4c) or CD4 KO (Fig. 4g) mice. Likewise, expression of granzyme B, a marker of CD8+ T cell activation, was increased after IT treatment with no differences between IT treated CD4+ CTRL and CD4 depleted (Fig. 4e) or CD4 KO (Fig. 4h) mice. Using *ex vivo* killing assays, we also observed that IT increased the lytic capacity of splenic CD8+ T cells across all groups (Fig. 4i,j). Not only was there no detriment in the killing function of the CD4 depleted (Fig. 4i) or CD4 KO (Fig. 4j) mice but they displayed a statistically significant improvement in killing function in comparison to IT-treated CD4+ CTRL mice. Similar results in regards to expression of NKG2D, IFNγ, and granzyme B as well as in vitro killing were observed in our long term CD4 depletion model (Figure S2).

**Figure 5:**
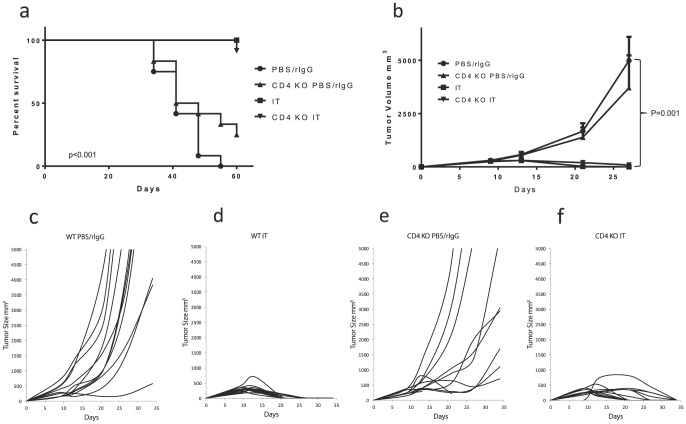
Anti-tumor effects of IT in CD4 knockout mice. 3LL tumor bearing WT or CD4 knockout (B6.129S2-CD4^tm1Mak^/J) mice were treated with IT or PBS/rIgG (control) and survival and tumor growth were measured. For *in vivo* tumor studies one million 3LL cells were administered by s.c. injection into the flank of C57BL/6 mice seven days prior to initiation of therapy. (**a**) Survival. (**b**) Mean tumor volume with SEM. (**c–f**) Growth plots of individual tumors in each group. N = 12 mice per group. (*P<.05, **P<.01, ***P<.001).

Importantly, the *in vivo* anti-tumor effects of IT were also not diminished in CD4 deficient mice. IT conferred long term survival to both CD4+ CTRL and CD4 KO mice bearing 3LL tumors whereas the median survival for untreated mice ranged from 41–44 days (Fig. 5a). Likewise, all tumor growth was abrogated in IT treated CD4+ CTRL and CD4 KO mice whereas most tumors displayed progressive outgrowth in vehicle treated mice (Fig. 5b–f). Similar results were seen in tumor-bearing mice in our CD4 depletion model (data not shown). Taken together with our data above, these results clearly indicate that, despite upregulation of exhaustion markers, the expansion, function, and anti-tumor effects of the bystander memory CD8+ T cells induced by IT are independent of CD4 help and in some aspects, possibly enhanced by CD4 deficiency.

### IL-21 signaling is not required for bystander memory CD8+ T cell expansion

IL-21 is able to sustain T cell responsiveness in chronic viral infections [27]. Other studies have found that a “helper-independent” phenotype of CTLs in cancer immunotherapy is due to IL-21 at the time of priming [28] and IL-21 has also been demonstrated to upregulate PD-1 [29]. Thus, IL-21 signaling could explain the phenotype and function of the expanded memory CD8 cells. Using mice lacking the receptor for IL-21 (IL-21rKO) the importance of IL-21 signaling on the IT induced function and expansion of bystander memory CD8+ T cells was examined. The lack of IL-21 signaling did not abrogate the IT induced proliferation of total CD8+ or CD44^high^ CD8+ memory cells in our models 11 days after the initiation of IT (Figure S3a,b). We also observed no differences in the expression of activation (NKG2D) or exhaustion (PD-1) markers on the CD44^high^ memory CD8 T cells (Figure S3c,d). These results demonstrate that bystander memory CD8+ T cell expansion and function does not require IL-21 signaling.

### Lack of CD4+ T cell help does not accelerate acute contraction of IT induced bystander memory CD8+ cells

**Figure 6:**
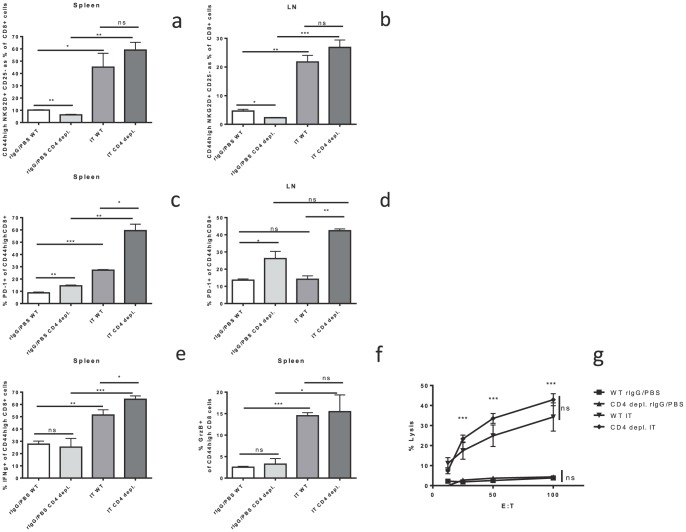
CD4 independence of IT induced bystander activated memory CD8+ T cells persists at longer time points. Control or long term CD4+ depleted C57BL/6 mice were treated with IT or PBS/rIgG (control) and harvested at Day 15 after the initiation of therapy. Bystander memory CD8+ T cells, PD-1 expression, interferon gamma production, and granzyme B production were quantified by flow cytometric analysis. Cytotoxic effector function was assayed by a redirected lysis assay. The percentage of CD8+ T cells with the CD44^high^ NKG2D+ CD25− bystander memory phenotype in the spleen (**a**) and LNs (**b**). PD-1 expression on the memory CD8+ T cells in the spleen (**c**) and LNs (**d**). Interferon gamma production (**e**) and granzyme B expression (**f**) in splenic memory CD8+ T cells. (**g**) Killing function of splenocytes from long term CD4 depleted mice expressed as percentage of maximal lysis. N = 3 mice per group (*P<.05, **P<.01, ***P<.001).

It has been reported that CD4 help is required for the maintenance and survival of memory CD8+ T cells [16]. Although lack of CD4 help does not appear to adversely affect the acute phase of the IT induced response, it is possible that the magnitude or duration of IT effects on bystander memory CD8+ T cells would be adversely affected leading to accelerated contraction or dysfunction. To answer this question we used our long term CD4 depletion model (Figure S1a) and examined the immune response at day 15 after the initiation of IT, as opposed to day 11 used above. Even at this later time point we see that the IT induced increase in bystander memory CD8+ T cells (CD25^−^NKG2D^+^CD44^high^) is maintained in CD4 depleted mice (Fig. 6a,b) as is the increase in PD-1 expression (Fig. 6c,d). Furthermore, the functionality of these cells as assayed by IFNγ (Fig. 6e), granzyme b (Fig. 6f), and *in vitro* killing (Fig. 6g) was also maintained at this time point despite lack of CD4+ help. This does not rule out the possibility that there could be increased contraction or dysfunction, at later time points, in CD4 deficient mice.

### CD4 depletion increases expression of PD-1 and conversion to effector phenotype on bystander memory CD8+ T cells after IT

**Figure 7:**
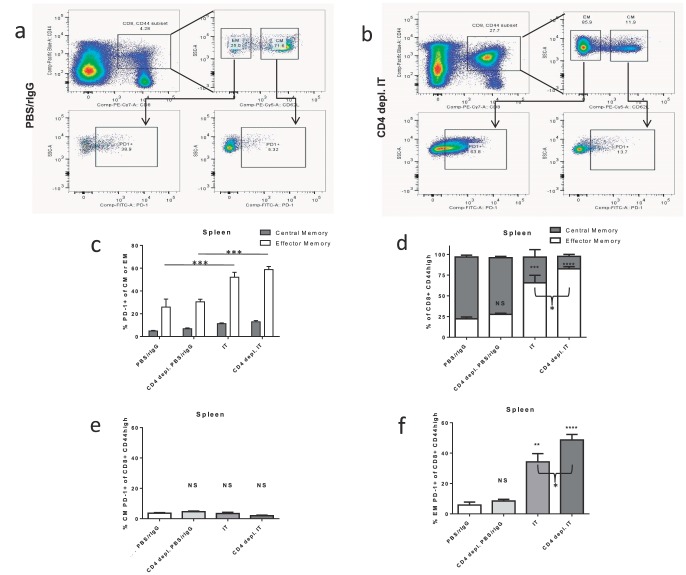
PD-1 expression on central and effector memory CD8+ T cells after IT in CD4+ T cell depleted mice. Control or CD4+ depleted C57BL/6 mice were treated with IT or PBS/rIgG (control). CD62L and PD-1 expression on memory T-cells was quantified by flow cytometric analysis 11 days after the initiation of IT. (**a,b**) Examples of the gating strategy for PD-1 expression on the CM and EM components of the memory CD8+ T cell compartment. The majority of cells in the memory compartment are CM in untreated mice (**a**) and EM in IT treated mice (**b**). (**c**) PD-1 expression on CM and EM cells. (**d**) The composition of the memory CD8+ T cell compartment in control or CD4 depleted mice treated with IT or PBS/rIgG. PD-1+ CM cells (**e**) and PD-1+ EM cells (**f**) as a percentage of the total memory CD8+ T cell compartment. Results are representative of two to three independent experiments with 3 mice per group (*P<.05, **P<.01, ***P<.001, ****P<.0001).

Based on the lack of functional exhaustion above, we hypothesized that upregulation of PD-1 corresponded with another defined role as a marker of effector T cells [30], [31]. Using CD62L to further characterize the bystander memory cells into central memory (CM) and effector/effector memory (EM) cells we investigated the expansion of these compartments and PD-1 expression 11 days after IT in CD4+ CTRL and CD4 deficient mice (Fig. 7a–c). In all cohorts of mice there was a significantly larger proportion of PD-1 expression on the EM CD62L^low^CD44^high^CD8+ cells than the CM CD62L^high^ CD44^high^CD8+ cells (26–59% vs. 5–13%; Fig. 7a–c). IT treated mice had a statistically significant increase in PD-1 expression of both CM and EM cells whereas CD4+ depletion did not effect PD-1 expression (Fig. 7c). There was also a shift in the composition of the memory compartment in IT treated mice from predominately CM to predominately EM with no more than 25% of memory cells displaying the EM phenotype in untreated mice and up to 75% after IT (Fig. 7d). Interestingly, in the setting of CD4 depletion, the IT induced shift to the EM phenotype was even more pronounced with 66% of memory cells from CD4+ CTRL mice displaying an EM phenotype compared to 83% in CD4 depleted mice (Fig. 7d, p = 0.07). Thus, IT increased PD-1 expression in the memory CD8+ T cells both by upregulating PD-1 expression on all memory cells and also by increasing the EM∶CM ratio. This effectively increased PD-1 expression since PD-1 expression was in general higher on EM cells (Fig. 7a–d). In contrast, CD4 depletion did not increase PD-1 expression outright on EM or CM cells (Fig. 7c) but further increased the EM∶CM ratio (Fig. 7d) thereby leading to the observed increase in PD-1 expression in the memory CD8+ T-cell compartment as a whole (Fig. 3). This notion, that PD-1 was predominately expressed on the EM cells and that lack of CD4 help increased the conversion of bystander cells from a central to effector memory phenotype, is further supported by examining PD-1 expression in the memory compartment as a whole. Neither IT nor CD4 depletion increased the subset of memory CD8+ T cells with a CM PD-1+ phenotype (Fig. 7e). Although IT did increase the percentage of PD-1+ CM cells (Fig. 7c), this was counterbalanced by the decrease of total CM cells in the memory CD8+ T cell compartment (Fig. 7d) leading to overall stability of CM PD-1+ cells as a percentage of total memory CD8+ T-cells. In contrast, the percentage of EM PD-1+ cells as a subset of total memory CD8+ T cells increased from 6% in CD4+ CTRL mice to 34% in IT treated CD4+ CTRL mice (Fig. 7f, p<0.01). CD4 depletion further increased this to 49% which was significantly more than IT treated CD4+ CTRL mice (Fig. 7f, p<0.05). It, therefore, seems likely that PD-1 upregulation in CD4 depleted mice is not a result of increased exhaustion as we previously suspected but rather due to differential regulation of the conversion of CM to EM in the absence of CD4 T cell help.

## Discussion

It has been demonstrated across different models that CD4+ help is needed to raise an efficient antigen specific CD8+ T cell response and for proper functioning of CD8+ memory cells. In general CD4+ T cell help can take many forms including activation of antigen presenting cells [32], [33], direct CD40-CD40L interactions [34], maintenance [14], [35] or mobilization of CD8+ cells [36]. In certain infections or inflammatory insults the initiation of CD8+ effector function may bypass the need for CD4+ help [35], [37], [38]. Even in these instances the central role of CD4+ help has been demonstrated for maintenance and memory functions [35], [38]. Some groups have demonstrated that CD4+ help is not required during priming but is mandatory for long term maintenance of the CD8+ cells [16] and protection from TRAIL induced death [15], while others have shown that CD8+ cells primed in the absence of CD4+ help may have an intact primary response but have a defective recall response even in the presence of CD4+ help [12]. The critical role of CD4+ T cell help in proper immune functioning is most clearly demonstrated in HIV+ patients suffering from AIDS. In models of viral infection including HIV it has been demonstrated that “helpless” CD8+ T cells upregulate PD-1 that diminishes the anti-viral response [17], [18]. In this setting PD-1 expression is associated with T cell exhaustion and disease progression [17]. Although the critical nature of CD4+ help in primary and memory CD8+ responses is well examined the nature and necessity of CD4+ help in bystander generated memory T-cell responses is less well defined.

We have previously demonstrated that IL-2 based immunotherapy, in combination with CD40 agonist, results in synergistic anti-tumor effects which are CD8+ T-cell dependent but not antigen restricted [3], [9] and (Fig. 1). There is a massive expansion of bystander (CD25−NKG2D+CD44^high^) memory CD8+ T-cells (Fig. 1) similar to “bystander expansion” that is seen after viral or bacterial infections [2], [3], [4], [10]. We have also demonstrated that IT can lead to progressive loss of CD4+ T cells by activation induced cell death [11]. Interestingly in our models of influenza infection these same bystander activated memory CD8+ T cells (CD25^−^NKG2D^+^CD44^high^) acutely expand and play an important role in controlling early viral infection in an antigen nonspecific manner [10]. Thus, understanding the role of CD4+ help in the expansion and effector function of these bystander cells has important implications for both cancer immunotherapy and viral immunity.

In this study we observed that lack of CD4+ cells causes homeostatic proliferation in the CD8+ T cell compartment of mice with increased numbers of CD8+ and CD44^high^CD8+ T cells in spleens and LNs of CD4 deficient non-IT treated mice compared to CD4 replete non-IT treated mice (Fig. 2a–d). This is in agreement with other studies of homeostatic expansion of CD8+ T-cells in CD4 deficiency. Using IT in models of CD4 deficient mice we found that surface expression of exhaustion marker PD-1 was upregulated (Fig. 3). However, in contrast to other settings of CD4 deficiency, the lack of CD4+ T cell help did not appear to have any implications on the expansion or function of bystander memory CD8+ T cells induced by IT. We found that neither the expansion of memory CD8+ T cells nor of the CD8+ T cell compartment in general was adversely affected by depletion or genetic disruption of CD4+ help (Fig. 2). Likewise, when analyzing the functional characteristics of these IT induced bystander memory cells, such as upregulation of NKG2D, increased production of granzyme b and interferon gamma, and *in vitro* killing, we observed no detrimental effects of CD4+ deficiency (Fig. 4). Importantly, the in vivo anti-tumor effects of IT also remained intact in CD4+ deficient models (Fig. 5). Although some of the phenotypic CD8+ T cell changes observed in other “helpless” models, such as upregulation of PD-1, were expressed, it appears that in our models the cells are not exhausted and that their functional effector capabilities remain intact. The long term consequences of IT in the setting of CD4 depletion on the function of CD8+ T-cells and response to tumor re-challenge have not been explored, however, we have previously demonstrated that even in the presence of CD4+ T-cell help secondary responses after immunotherapy can become impaired due to activation induced cell death after strong cytokine stimulation [11].

In some aspects the expansion and effector function of CD8+ T cells after IT was enhanced in our CD4 deficient models. Whether our results are due to lack of helper CD4+ T cells or other CD4 expressing cells such as regulatory T cells or natural killer-T cells is unclear. Indeed one general shortcoming of our models is that they fail to distinguish between CD4+ helper T-cells and other CD4+ cell types. These findings could be attributed to lack of CD4+CD25+FoxP3+ regulatory T cells (Tregs) in the CD4 deficient models. However, the effects of IT in CD25 deficient mouse models (data not shown) were similar to that observed in control mice and did not mirror the enhanced effects in CD4 deficient models. This would argue against lack of Tregs as the mechanism for enhanced response seen in CD4 deficient models although further study in other models is needed. Likewise, the increased expression of PD-1 seen after IT (Fig. 3) in our CD4 deficient mice was not mirrored in our CD25 deficient mouse models (data not shown) also arguing against lack of Tregs as the underlying mechanism for this finding. The increased shift from CM to EM phenotype in CD4 deficient models (Fig. 7) could explain the increased killing function after IT but does not explain the increased expansion. As previously mentioned, there is increased baseline homeostatic expansion of the CD8+ compartment even in the absence of IT and it is reasonable to presume that this could contribute to the increased expansion seen with IT.

Although PD-1 is generally considered an exhaustion marker, other functions have also described. For example PD-1 has been shown to be upregulated during activation it can have a role in stimulating CD8+ cells during a primary response, and it can be a marker of cytotoxic effector function [30], [31]. The exact reason for the discrepancy between the need for CD4+ help and the role of PD-1 in our models versus much of the published literature is unresolved. A few possibilities might explain this discrepancy: 1) IL-21 in the setting of IT and anti-tumor responses bypasses the need for CD4+ help; 2) the effects of our highly stimulatory IT are such that they “rescue” the “helpless” CD8+ cells from exhaustion; or 3) the regulation of bystander activated memory CD8+ T-cells is different than the regulation of antigen specific CD8+ T-cells. Further study is needed to resolve these possibilities although our current findings, highlighted below, suggest that these last two possibilities are most likely.

PD-1 has been described to be inducible on many subsets of immune cells [26] and on CD8+ T cells can occur as early as 24 hours after stimulation [39]. The role of early PD-1 upregulation is not yet completely defined, but it is likely that it dampens CD8+ effector responses to control the immune response. Prolonged upregulation of PD-1 is associated with exhaustion [17]. Thus, the CD4+ independent effects of IT may be an artifact of our model and at longer time points there could be increased exhaustion and dysfunction or acceleration in the contraction phase post-IT. To address this possibility we examined the effects of long term depletion of CD4+ T cells on IT and also analyzed later time points after the completion of IT. 15 days after the initation of IT there was no deterioration of the numbers or function of the bystander memory CD8+ T cells in CD4 depleted mice as compared to IT treated CD4+ CTRL mice (Fig. 6). This does not rule out the possibility that there could be increased contraction or dysfunction, at even later time points, in CD4 deficient mice. We have previously observed that there are negative long-term consequences after IT even in the presence of CD4+ T-cell help [11].

Others have also reported CD4 helper independent CD8+ T-cell anti-tumor activity. In a report by Adam et al. [40] they find that in certain settings NK cells can activate DCs and IL-12 production bypassing the need for CD4 help in the activation of anti-tumor CD8+ T-cells. We have previously demonstrated that depletion of NK cells does not abrogate the efficacy of our IT making it unlikely that this mechanism is responsible for our findings [9]. Another possibility is that the CD40 agonist anti-body and may bypass the need for CD4+ help by directly activating APCs [41] which seems plausible. It has also been demonstrated in cancer immunotherapy models that there can be a “helper-independent” phenotype of CTLs after immunotherapy that is due to IL-21 at the time of priming [28]. IL-21 has been described to be predominately produced by CD4 T cells and natural killer cells [42]. It promotes CD8 cell activation and has been proposed to act as a third signal [27], [43]. IL-21 has also been demonstrated to upregulate PD-1 [29]. Thus, it is possible that IL-21 present in the setting of immunotherapy, but perhaps lacking in antigen specific CD8+ T cell expansion models, is circumventing the need for CD4+ help in this setting. Our results, however, demonstrated that genetic abrogation of IL-21 signaling did not disrupt the phenotype of the CD8+ bystander memory cells in our model (Figure S3). This argues against IL-21 signaling as being the sole or predominant mechanism of the “helper-independent” phenotype. This does not discount the possibility of other potential differences between the immune system in IT settings and other settings could be responsible for our findings. The upregulation of PD-1 on “helpless” CD8+ cells has been shown to be marker of the exhaustion and dysfunction of T cells. In our studies, however, we found no evidence of dysfunction or exhaustion in the CD4 deficient setting and found that in some instances there was even upregulation of functional activity. Further examination showed that in the CD8+ memory compartment PD-1 expression was much higher on effector memory than central memory cells. Although PD-1 increased in response to IT, the additional increase in PD-1 seen in CD4 deficient mice was due to an increased shift from the central to effector memory phenotypes compared to CD4 replete mice treated with IT (Fig. 7). This increase in effector memory cells could also explain the enhanced effectiveness of IT in CD4 deficient models seen in some of our studies.

Taken together our results suggest that, in contrast to antigen-specific CD8+ T cell expansion, CD4+ T cell help is not necessary for the expansion and activation of antigen-nonspecific bystander memory CD8+ T cells following immunotherapy, yet may play a role in regulating the conversion of these bystander cells from a central memory to effector phenotype. Further study is needed to clarify whether bystander cells induced by acute viral infections are regulated in a similar manner. Another important consideration, which is a topic of current investigation in our lab, is how the altered function and regulation of bystander activated memory CD8+ T cells effects the functioning of the rest of the immune system. The strong immunostimulatory conditions, such as IL-2 immunotherapy or viral infection, that induce bystander activated memory CD8+ T cells may allow for expansion and antigen independent effector function of these cells, even in the absence of CD4+ help which is lost via activation induced cell death. What effect these conditions have on the priming of a new response is unknown. However, we could posit, and are currently investigating, that the epitope spreading required for a successful immune response in the setting of viral infection or cancer immunotherapy could be hampered. Also under investigation is the long term effects of IT induced bystander cells on immune function weeks to months after post-IT contraction. These unresolved questions will provide us with greater insight into the functioning, regulation, and consequences of antigen non-specific bystander memory CD8+ T cell responses.

## Supporting Information

Figure S1 **Depiction of treatment schemas and evaluation of CD4 deficient models.** (**a**) Schema of short-term CD4 depletion, long term CD4 depletion, and immunotherapy protocols. These therapies were administered to naïve or tumor bearing mice depending on the experiment. (**b,c**) Evaluation of the CD4^+^ T-cell compartment in control and CD40 + IL-2 immunotherapy (IT) treated mice in WT or CD4 deficient mice. Lymph nodes (**b**) and spleens (**c**) of anti-body depleted or knock out mice were evaluated for CD4+ staining by FACS analysis after treatment with vehicle or IT. Data are results of two to four independent experiments with three mice per group. (*** *P*<.001).
(TIF)

Figure S2 **IT induced immunologic effects in the long term CD4 depletion model.** Control or long term CD4 depleted C57BL/6 mice were treated with IT or PBS/rIgG (control) and effects on CD8+ T cell expansion, PD-1 expression, and function were quantified by flow cytometric analysis. NKG2D and granzyme B expression were quantified by flow cytometric analysis. Interferon gamma production was quantified by flow cytometric analysis after *in vitro* restimulation of splenocytes with PMA/Ionomycin (0.16/1.6 ug/ml) for one hour followed by incubation with golgi stop (0.7 ug/ml) for three hours. CD8+ T cell killing function was assayed by scintillation counting using an *in vitro* redirected lysis assay with ^51^Cr labeled P815 target cells incubated for 30 minutes with 10 ug/mL anti-CD3e. CD8+ (**a,b**) and memory CD8+ (**c,d**) T cell numbers in the LNs (**a,c**) and spleens (**b,d**) of control or CD4+ T cell depleted mice treated with vehicle or IT. Number of PD-1+ memory (CD44^high^) CD8+ T cells in LNs (**e**) and spleen (**f**) of IT or vehicle treated mice. (**g**) NKG2D expression, (**h**) Interferon gamma production, and (**i**) Granzyme B expression by memory CD8+ T cells in long term CD4 depleted mice. (**j**) Killing function of splenocytes from CD4 depleted mice expressed as percentage of maximal lysis. Results are representative of three independent experiments with a minimum of three mice per group. (*P<.05, **P<.01, ***P<.001).
(TIF)

Figure S3 **IT induced function and expansion of bystander memory CD8+ Tcells does not require IL-21 signaling.** Wild-type or IL-21 RKO were treated with IT or PBS/rIgG (control) and harvested at Day 11 after the initiation of therapy. CD8+ T cell and memory CD8+ T cell expansion, and NKG2D and PD-1 expression were quantified by flow cytometric analysis. Total number of splenic CD8+ T cells (**a**) and memory CD8+ T cells (**b**). The percentage of NKG2D+ (**c**) or PD-1+ (**d**) splenic memory CD8+ T cells. Results are representative of three independent experiments with three mice per group. (*P<.05, **P<.01, ***P<.001).
(TIF)
