# Supplementary figures and images for: Bystander Activation and Anti-Tumor Effects of CD8+ T Cells Following Interleukin-2 Based Immunotherapy Is Independent of CD4+ T Cell Help
Source: PLoS One. 2014 Aug 13;9(8):e102709. doi: 10.1371/journal.pone.0102709 (PMC4131875; doi:10.1371/journal.pone.0102709)

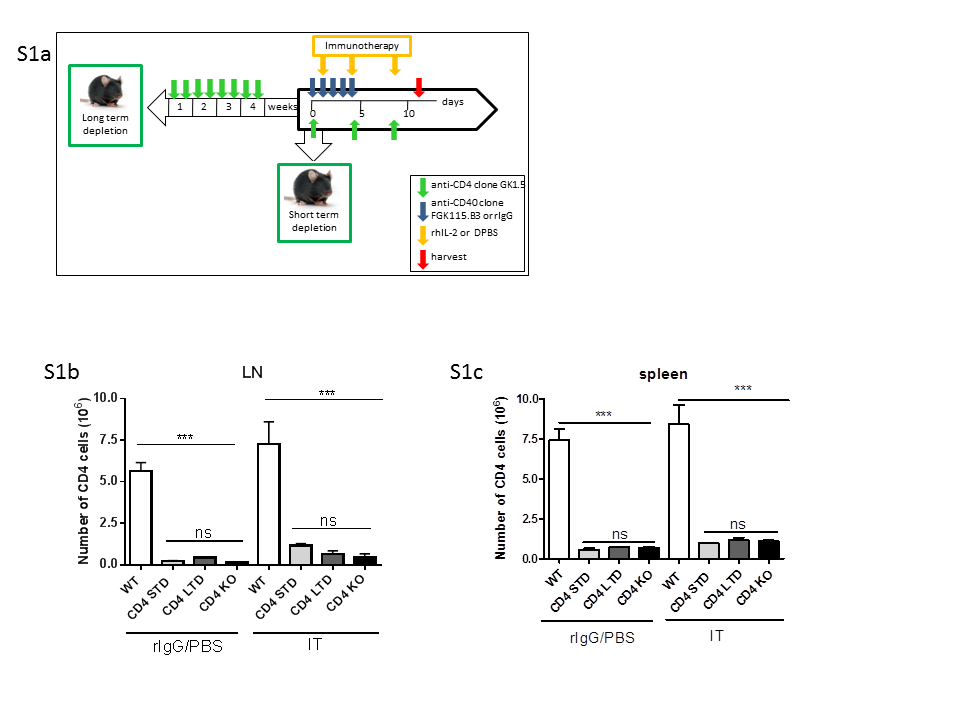

Supplement: Figure S1 — Depiction of treatment schemas and evaluation of CD4 deficient models. (a) Schema of short-term CD4 depletion, long term CD4 depletion, and immunotherapy protocols. These therapies were administered to naïve or tumor bearing mice depending on the experiment. (b,c) Evaluation of the CD4+ T-cell compartment in control and CD40 + IL-2 immunotherapy (IT) treated mice in WT or CD4 deficient mice. Lymph nodes (b) and spleens (c) of anti-body depleted or knock out mice were evaluated for CD4+ staining by FACS analysis after treatment with vehicle or IT. Data are results of two to four independent experiments with three mice per group. (*** P<.001). (TIF) [file pone.0102709.s001.tif]

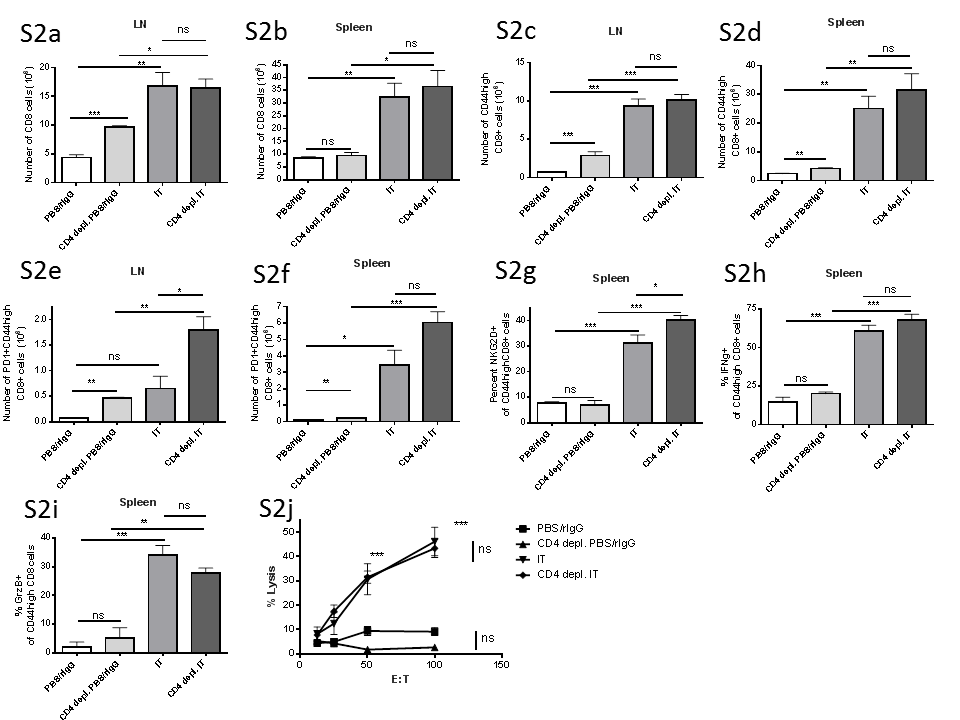

Supplement: Figure S2 — IT induced immunologic effects in the long term CD4 depletion model. Control or long term CD4 depleted C57BL/6 mice were treated with IT or PBS/rIgG (control) and effects on CD8+ T cell expansion, PD-1 expression, and function were quantified by flow cytometric analysis. NKG2D and granzyme B expression were quantified by flow cytometric analysis. Interferon gamma production was quantified by flow cytometric analysis after in vitro restimulation of splenocytes with PMA/Ionomycin (0.16/1.6 ug/ml) for one hour followed by incubation with golgi stop (0.7 ug/ml) for three hours. CD8+ T cell killing function was assayed by scintillation counting using an in vitro redirected lysis assay with 51Cr labeled P815 target cells incubated for 30 minutes with 10 ug/mL anti-CD3e. CD8+ (a,b) and memory CD8+ (c,d) T cell numbers in the LNs (a,c) and spleens (b,d) of control or CD4+ T cell depleted mice treated with vehicle or IT. Number of PD-1+ memory (CD44high) CD8+ T cells in LNs (e) and spleen (f) of IT or vehicle treated mice. (g) NKG2D expression, (h) Interferon gamma production, and (i) Granzyme B expression by memory CD8+ T cells in long term CD4 depleted mice. (j) Killing function of splenocytes from CD4 depleted mice expressed as percentage of maximal lysis. Results are representative of three independent experiments with a minimum of three mice per group. (*P<.05, **P<.01, ***P<.001). (TIF) [file pone.0102709.s002.tif]

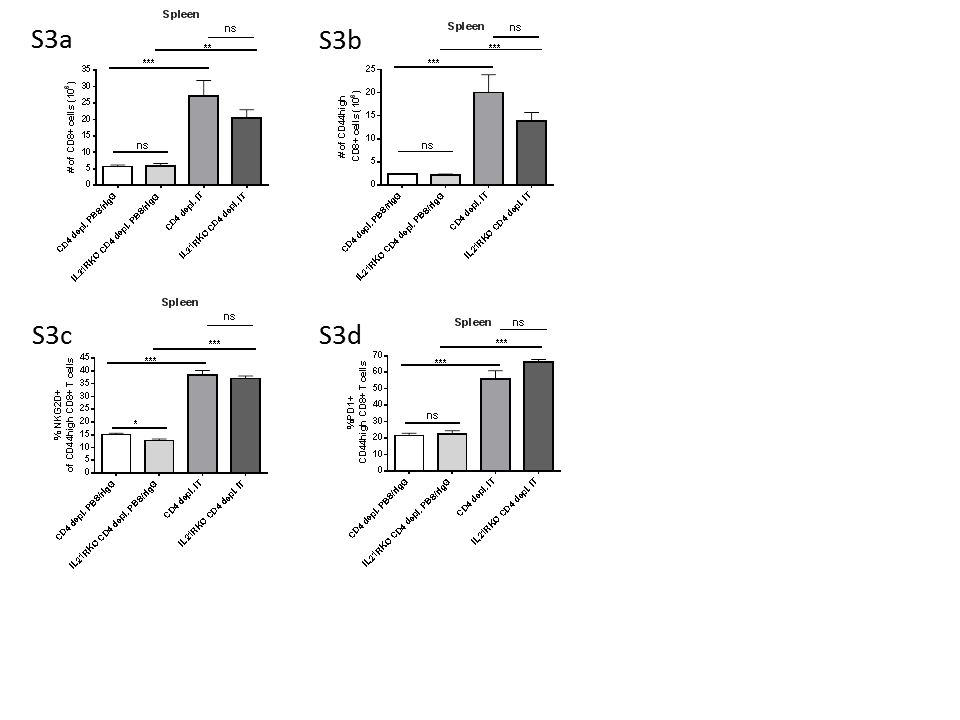

Supplement: Figure S3 — IT induced function and expansion of bystander memory CD8+ Tcells does not require IL-21 signaling. Wild-type or IL-21 RKO were treated with IT or PBS/rIgG (control) and harvested at Day 11 after the initiation of therapy. CD8+ T cell and memory CD8+ T cell expansion, and NKG2D and PD-1 expression were quantified by flow cytometric analysis. Total number of splenic CD8+ T cells (a) and memory CD8+ T cells (b). The percentage of NKG2D+ (c) or PD-1+ (d) splenic memory CD8+ T cells. Results are representative of three independent experiments with three mice per group. (*P<.05, **P<.01, ***P<.001). (TIF) [file pone.0102709.s003.tif]
